# *In Vivo* Turnover of Tau and APP Metabolites in the Brains of Wild-Type and Tg2576 Mice: Greater Stability of sAPP in the β-Amyloid Depositing Mice

**DOI:** 10.1371/journal.pone.0007134

**Published:** 2009-09-22

**Authors:** Jose Morales-Corraliza, Matthew J. Mazzella, Jason D. Berger, Nicole S. Diaz, Jennifer H. K. Choi, Efrat Levy, Yasuji Matsuoka, Emmanuel Planel, Paul M. Mathews

**Affiliations:** 1 Nathan Kline Institute for Psychiatric Research, Orangeburg, New York, United States of America; 2 New York University School of Medicine, Orangeburg, New York, United States of America; 3 Department of Neurology, Georgetown University Medical Center, Washington, D. C, United States of America; 4 Centre Hospitalier de l'Université Laval, Neurosciences, Québec, Canada; Mental Health Research Institute of Victoria, Australia

## Abstract

The metabolism of the amyloid precursor protein (APP) and tau are central to the pathobiology of Alzheimer's disease (AD). We have examined the *in vivo* turnover of APP, secreted APP (sAPP), Aβ and tau in the wild-type and Tg2576 mouse brain using cycloheximide to block protein synthesis. In spite of overexpression of APP in the Tg2576 mouse, APP is rapidly degraded, similar to the rapid turnover of the endogenous protein in the wild-type mouse. sAPP is cleared from the brain more slowly, particularly in the Tg2576 model where the half-life of both the endogenous murine and transgene-derived human sAPP is nearly doubled compared to wild-type mice. The important Aβ degrading enzymes neprilysin and IDE were found to be highly stable in the brain, and soluble Aβ40 and Aβ42 levels in both wild-type and Tg2576 mice rapidly declined following the depletion of APP. The cytoskeletal-associated protein tau was found to be highly stable in both wild-type and Tg2576 mice. Our findings unexpectedly show that of these various AD-relevant protein metabolites, sAPP turnover in the brain is the most different when comparing a wild-type mouse and a β-amyloid depositing, APP overexpressing transgenic model. Given the neurotrophic roles attributed to sAPP, the enhanced stability of sAPP in the β-amyloid depositing Tg2576 mice may represent a neuroprotective response.

## Introduction

The brain accumulation of abnormal proteins is a common hallmark of multiple neurodegenerative diseases. Alzheimer's disease (AD) brains are characterized by extracellular aggregates of the small (40 and 42 residues) β-amyloid peptide (Aβ) [1], and intraneuronal neurofibrillary tangles, composed of hyperphosphorylated tau assembled in paired helical filaments [2]. Aβ is generated by proteolysis of the ∼100-kDa amyloid precursor protein (APP), a broadly expressed type-1 transmembrane protein that is found primarily in the trans-Golgi network, endocytic compartments, and at the cell surface [3]. β-Cleavage of APP, mediated by BACE1, occurs within the luminal/extracellular domain of APP and generates two APP fragments: a large, soluble amino-terminal fragment (sAPPβ) that is secreted from the cell and a transmembrane, carboxyl-terminal fragment (βCTF) containing the whole Aβ peptide that remains associated with the cell [4]. An alternative pathway involves the cleavage of APP 16 residues downstream of this site at the α-cleavage site, which is mediated by metalloproteases [5]. Like β-cleavage, α-cleavage generates an sAPP fragment (sAPPα) that is secreted from the cell and an αCTF that remains membrane-associated. α-Cleavage occurs within the Aβ peptide sequence and as such prevents the generation of Aβ from a given APP molecule. Aβ is generated from the βCTF by an intramembrane cleavage (mediated by the presenilin γ-secretase complex) that occurs primarily at 40 residues, and to a lesser extent at 42 residues, downstream from the β-cleavage site, releasing Aβ1–40 and Aβ1–42 [4].

While much is known about the proteolytic processes that lead to the generation of these APP metabolites, their subsequent metabolism and function(s) in the central nervous system (CNS) are not as well defined [6]. APP that is not cleaved at the α- or β-sites can be degraded through the lysosomal pathway [7]–[9]. Aβ accumulation in the brain during AD is dependent on the misbalance of production and turnover of this peptide, with neprilysin and insulin degrading enzyme (IDE) known to be important Aβ degrading enzymes in the brain [10], [11]. The turnover of the secreted sAPP fragments *in vivo* has not been extensively examined [12], and, unlike APP, cannot be assessed using cell culture systems where sAPP is simply secreted into the growth media. Understanding the turnover of proteins in the brain that play a central role in neurodegenerative diseases, such as APP and tau, can help to explain why a protein/peptide fragment, such as Aβ, accumulates. Additionally, understanding the *in vivo* turnover of APP metabolites and tau in the normal, intact brain is an important consideration when developing therapeutic strategies directed at altering their levels. In the case of APP, the fragments that are generated may have important physiological functions related to their abundance and regulation in the CNS. For example, sAPP is thought to have neuroprotective and neurotrophic functions [13], [14], which would be unique to the CNS and dependent upon its stability in the brain. In order to directly examine the turnover of endogenous APP, APP metabolites, and tau in an intact brain, we have blocked *de novo* protein synthesis in mice and examined the stability of these proteins over time.

## Results

**Figure 1:**
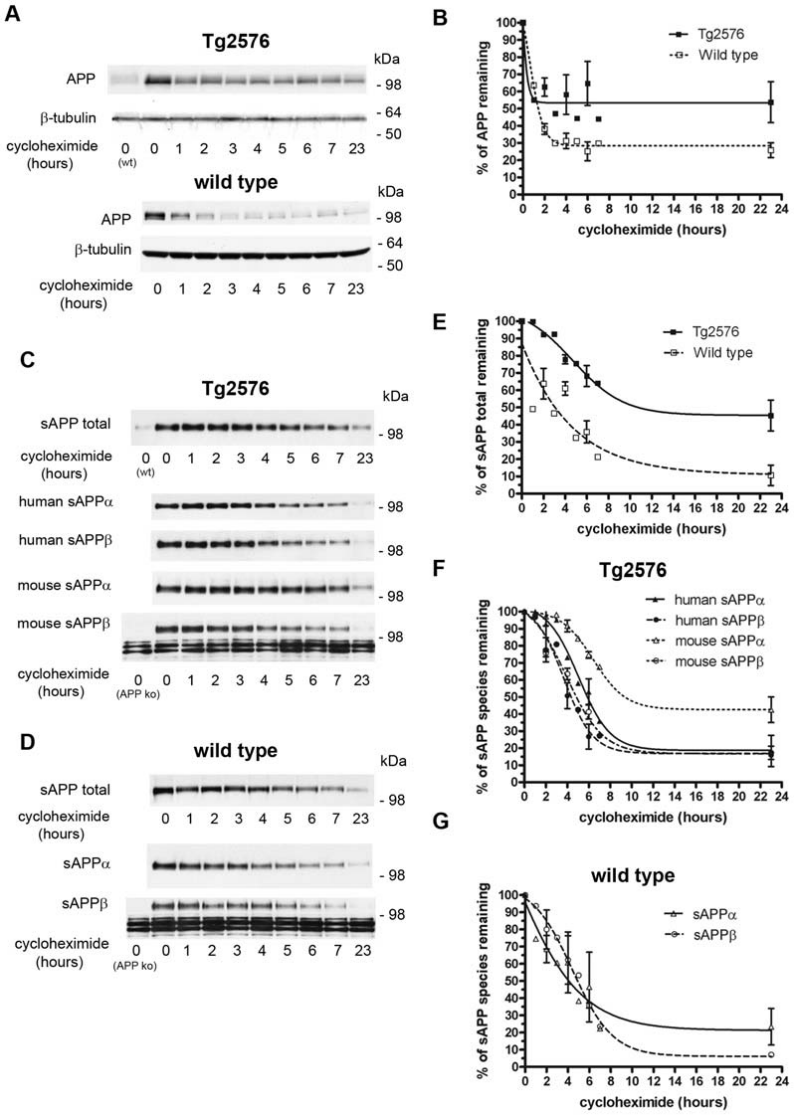
Turnover of APP and sAPP in 16-month-old Tg2576 mouse and wild-type brains following cycloheximide treatment. Western blotting is shown of total proteins isolated from brain tissue following cycloheximide treatment of mice for the indicated times. (A) Transgene-derived human and endogenous APP in Tg2576 mice and endogenous murine APP in wild-type mice was detected with the anti-C-terminal APP antibody C1/6.1; β-tubulin, which is known to be stable in the CNS [52], is shown in control Western blots. An untreated wild-type mouse (wt) is included in the Tg2576 analysis to show the increased APP expression in this model. (B) Quantification of APP turnover in the Tg2576 and wild-type mice normalized to the band density without cycloheximide treatment; two-way ANOVA was used to compare APP turnover in the Tg2576 and wild-type mice (p<0.001). (C) Following the indicated cycloheximide treatment times, sAPP total was detected with 22C11 in Tg2576 mice. Human sAPPα and sAPPβ were detected by Western blotting with 6E10 and 6A1, respectively, and endogenous murine sAPPα and sAPPβ were detected by Western blotting with m3.2 and 242, respectively. Non-specific bands detected by the affinity purified polyclonal 242 are shown by the APP knockout brain homogenate. (D) Levels of endogenous sAPP total, sAPPα and sAPPβ were detected by Western blotting in wild-type mice following cycloheximide treatment as described above. The graphic representations in (E) compare the quantification of the levels of sAPP total in Tg2576 and wild-type mice (p<0.001). In (F), the turnover of the indicated sAPP species in the Tg2576 mice is shown (p<0.001, comparing murine sAPPα to the other sAPP species). In (G), the levels of endogenous sAPPα and sAPPβ in wild-type mice is shown (p>0.5). Throughout, quantifications are from two experiments (mean ± SEM) as specified in the Methods section.

Following treatment with cycloheximide, we examined by Western blotting APP holoprotein levels in the brains of Tg2576 mice with β-amyloid deposits and wild-type littermates. Figure 1A and 1B show that APP levels rapidly declined in Tg2576 and wild-type mice following protein synthesis inhibition. The initial turnover of the human Swedish APP in the Tg2576 model, which is overexpressed at ∼6-fold of the endogenous protein ([15]; see also Figure 1A), is similar to the turnover of the endogenous APP in a wild-type mouse, with ∼40% of the pre-existing APP degraded within the first hour. In both Tg2576 and wild-type mice, a portion of the initial APP was found to persist up to 23 hours following the inhibition of protein synthesis, with a greater amount of the initial APP pool persisting in the Tg2576 mice (∼50%) than in the wild-type mice (∼30%); p<0.001, two-way ANOVA comparing the APP turnover in the Tg2576 and wild-type mice.

Next, we examined the levels of sAPP in the brain following cycloheximide treatment. Using 22C11 antibody to detect all sAPP fragments, human and mouse and following cycloheximide treatment, it was found that sAPP total is cleared from the brain in the Tg2576 (top panel of Figure 1C) and wild-type mice (top panel of Figure 1D). The half-life for sAPP total was found to be more than 7 hours in Tg2576 mice and ∼4 hours in wild-type mice (calculated as a first-order process [16]) (Figure 1E); p<0.001, two-way ANOVA comparing the sAPP total turnover in the Tg2576 and wild-type mice. Initial sAPP total levels in the brain of Tg2576 mice were increased relative to wild type, as is expected given increased APP expression (see top panel Figure 1C). Moreover, sAPP itself is more abundant in mouse brain than APP (see Figure S1, lower panel E), consistent with the sAPP species having a longer half-life in the brain than APP.

To further explore potential differences in the *in vivo* half-life of sAPP, we differentiated in the Tg2576 mice sAPPα from sAPPβ, both human and murine (Figure 1C). In the Tg2576 mice, the turnover of murine sAPPα (sAPPα t½ >7 hours) was found to be slower than the turnover of murine sAPPβ, human sAPPα, and human sAPPβ (Figure 1F; t½ ∼5–6 hours) (p<0.001, two-way ANOVA comparing murine sAPPα to the other sAPP species), although the relative stability of all sAPP species in the Tg2576 mouse brain is in agreement with the slow turnover of sAPP total seen in this model (Figure 1E). In wild-type mice (Figure 1D), both the endogenous murine sAPPα and sAPPβ were found to have *in vivo* half-lives of ∼4 hours (Figure 1G), in close agreement with the turnover seen for sAPP total detected with 22C11 in the wild-type mice (Figure 1E). No breakdown products were specifically detected by the antibody against APP (C1/6.1) or the different sAPP antibodies in these Western blot analyses.

**Figure 2:**
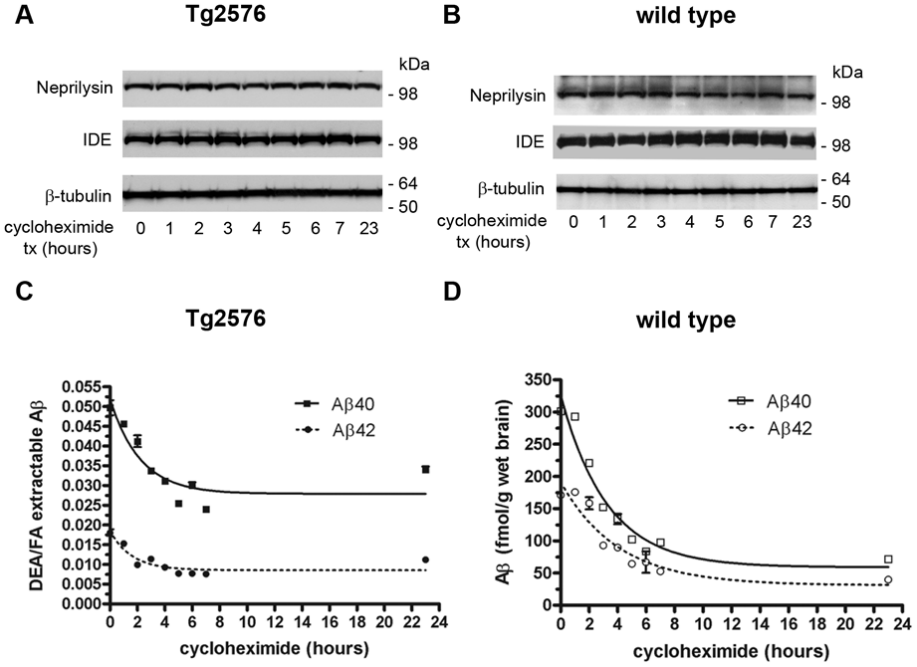
Turnover of neprilysin, IDE and Aβ levels in 16-month-old Tg2576 and wild-type mouse brains following cycloheximide treatment. Neprilysin and IDE turnover is shown by Western blotting in Tg2576 (A) and wild-type mice (B); β-tubulin is shown as a control. (C) The ratio of DEA-extractable to formic acid-extractable human Aβ40 and Aβ42 in Tg2576 mice were measured by sandwich ELISA at the indicated times following cycloheximide treatment (mean ± SEM). (D) Turnover of endogenous DEA-extractable Aβ40 and Aβ42 levels in wild-type mice (mean ± SEM).

Prior to examining the clearance of Aβ in the brain, we determined the stability of two Aβ degrading enzymes: neprilysin and insulin degrading enzyme (IDE). The levels of these important Aβ degrading enzymes do not decline rapidly following cycloheximide treatment in either Tg2576 mice (Figure 2A) or wild-type mice (Figure 2B), and both proteases were readily detected following 23 hours of cycloheximide treatment. Plaque-associated Aβ is thought to be highly stable within the brain [17]. Total Aβ levels in the Tg2576 mice, determined by formic acid extraction of plaque Aβ, varied between animals independently of cycloheximide treatment time (data not shown). However, the pool of soluble Aβ measured by DEA extraction normalized to formic-acid extractable Aβ in a given Tg2576 mice was found to rapidly decrease following cycloheximide treatment (Figure 2C). The soluble Aβ pool in the Tg2576 mice decreased by ∼50% at maximum, suggesting that soluble Aβ in the β-amyloid depositing mice may be in equilibrium with plaque-associated Aβ. We also examined DEA extractable, soluble endogenous Aβ levels in wild-type mice (Figure 2D). Brain Aβ40 and Aβ42 levels decreased rapidly, at a rate similar to that of soluble Aβ in the Tg2576 mice, and consistent with the rapid depletion of precursor APP in the brain. In comparison to the Tg2576 mice, less of the initial Aβ remained after 23 hours of cycloheximide treatment in the wild-type mice.

**Figure 3:**
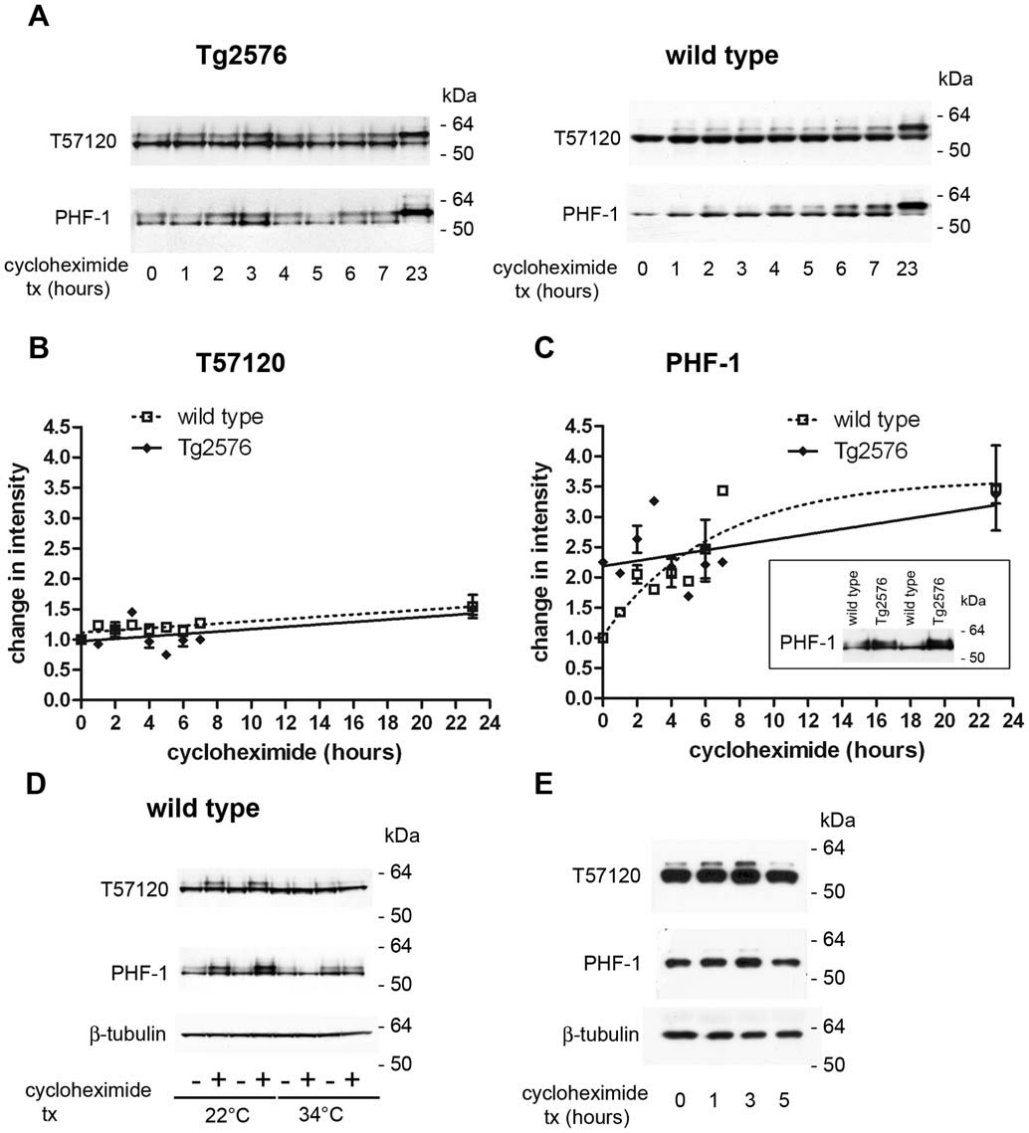
Metabolism of tau following protein synthesis inhibition. (A) Western blots are shown using an antibody that recognizes tau independent of its phosphorylation status (T57120, top panels) and an antibody that detects a phospho-epitope on tau (PHF-1, bottom panels) in Tg2576 mouse brain and in wild-type mouse brain. Total brain proteins were analyzed as in Figure 1. (B) Change in the intensity of the T57120 signal following cycloheximide treatment in Tg2576 and wild-type mice is shown (mean ± SEM). (C) Change in the intensity of the PHF1 signal following cycloheximide treatment in Tg2576 and wild-type mice is shown (mean±SEM). Signal intensity is normalized to the PHF1 signal in wild-type mice without treatment (inserted Western blot). (D) Tau levels detected with T57120 and PHF-1 antibodies in wild-type mouse brain following cycloheximide treatment when mice were housed at ambient temperature (22°C) or at 34°C for 19 hours. β-tubulin is shown as a loading control. (E) Tau levels in primary neurons were detected by Western blotting with T57120 and PHF-1 antibodies after cycloheximide treatment as indicated. β-tubulin is shown as a loading control.

Finally, we determined the *in vivo* turnover rate of tau. In Tg2576 mice, the levels of tau protein (T57120) were unchanged with cycloheximide treatment over 23 hours. Similarly, in the wild-type mice levels of tau protein were unchanged following protein synthesis inhibition (Figure 3A and 3B). In contrast, a greater upward shift in the mobility of tau in the wild-type mice compared to the Tg2576 mice following cycloheximide treatment is suggestive of a robust increase in tau phosphorylation. Consistent with this idea, the levels of phosphorylated-tau detected by PHF-1 dramatically increased in the wild-type mice following protein synthesis inhibition (∼3.4-times at 23 hours), whereas the change in PHF-1 signal from baseline in the Tg2576 mice following cycloheximide treatment was less (∼1.5-times at 23 hours) (Figure 3A and 3C). While the initial PHF-1 signal was less in the wild-type mice compared to the Tg2576 mice (Tg2576 ∼2.2-times wild-type without treatment), after 23 hours of protein-synthesis inhibition the level of PHF-1 reactivity was similar in both groups of mice (Figure 3C). Cycloheximide treatment is known to cause hypothermia [18], and a decrease in body temperature in mice increases the levels of phospho-tau [19], [20]. To determine whether cycloheximide-treatment increases tau phosphorylation in the wild-type mice primarily as the result of a decrease in body temperature, mice housed at room temperature were compared to mice housed at 34°C. When housed for 19 hours at 22°C following cycloheximide treatment body temperature decreased by 4.8+/−1.1°C (n = 6), whereas mice housed at 34°C showed a smaller drop in body temperature (2.3+/−0.4°C; n = 6). Control mice showed no change in body temperature when housed at 34°C. When compared by Western blotting, the mice housed at 34°C had less of an increase in phospho-tau levels following cycloheximide treatment (Figure 3D). Primary neurons maintained at 37°C showed a small and transient increase in phospho-tau following cycloheximide treatment that was also reflected in the appearance of a slower migrating band in the total tau Western blot, possibly due to differential turnover of the relevant tau kinases and phosphatases (Figure 3E). Nevertheless, the reduction in cycloheximide-dependent tau phosphorylation *in vivo* when body temperature is maintained suggests that the bulk of the increase in tau phosphorylation over a longer period results from a decrease in body temperature (Figure 3D).

## Discussion

Global protein-synthesis inhibition can be used to determine the stability of proteins in an animal, and is appealing when examining protein turnover in the CNS as the protein-synthesis inhibitor cycloheximide readily passes the blood-brain barrier and the conditions for CNS-protein-synthesis inhibition in mice have been well established [21]. Using this, we examined in the brain the metabolic turnover of two proteins key to AD pathobiology, tau and APP, comparing wild-type mice with a transgenic β-amyloid depositing AD mouse model Tg2576 [15]. The turnover for a metabolite such as the secreted sAPP species as well as extracellular Aβ cannot be extrapolated from a cell culture system, and can only be determined in the intact brain. Moreover, determining the impact of plaque pathology and associated gliosis [22] on the turnover rate for tau, APP and other APP metabolites such as sAPP also necessarily requires an intact *in vivo* system.

Our finding that tau is a highly stable protein in the brains of both wild-type and Tg2576 mice is in agreement with a report of tau protein persisting for more than 30 days during transport along the mouse optic nerve from the retina [23], and suggests that tau remains long-lived once delivered to the distal neurite. In both wild-type and Tg2576 mice, the total levels of tau protein were unchanged with as long as 23 hours of cycloheximide treatment, and this stability of tau *in vivo* may predispose it to accumulation such as occurs in AD with paired helical filament tau [24], [25]. Unexpectedly, both in wild-type and Tg2576 mice the levels of phosphorylated-tau increased following inhibition of protein synthesis, apparently due to hypothermia. Hypothermia is a powerful enhancer of tau phosphorylation through its effects on the balance of kinase and phosphatase activities [19], [20], although this effect was less pronounced in the Tg2576 mice, possibly because baseline phospho-tau is higher in these mice [26]–[28].

The rapid turnover of endogenous APP following protein synthesis inhibition in the brain is consistent with what has been reported by pulse-chase of cultured rodent neurons and glia [29]. APP is transported by rapid anterograde transport in neurons, with most of its processing apparently occurring at the distal neurite [30]. Following metabolic labelling of retinal ganglion cells, Lyckman *et al*. [31] found that APP, once transported to the presynaptic termini, had a half-life of less than 4 hours. Consistent with these reports, our findings present a global picture of APP metabolism in the brain showing a rapid bulk-turnover of the holoprotein, both under normal expression levels in wild-type mice and with APP overexpression and plaque pathology in aged Tg2576 mice. Indeed, in spite of the greater APP expression in the transgenic mice the initial turnover of APP is similar in Tg2576 and wild-type mice, although a greater proportion of APP stably persists following extended cycloheximide treatment in the APP overexpressing transgenics.

The bulk flow of the CSF in a mouse brain, measured by inulin [32] or albumin [33] transport, is approximately 2–3 hours, which is similar to the turnover of sAPP in a wild-type mouse brain and suggests that this soluble APP metabolite may diffuse in the brain along with the bulk flow of CSF as a major pathway for its catabolism [16]. However, the turnover of sAPP in the brain was found to be slower in the Tg2576 mouse than in a wild-type animal. sAPP has been shown to support cell survival, neurite outgrowth, synaptogenesis, synaptic plasticity, can modify the response of neurons to injury, and can substantially rescue CNS deficits in an APP null mouse [34]–[39]. The increased stability of sAPP in Tg2576 mice relative to wild-type mice is consistent with a greater need for neurotrophic support in the β-amyloid containing brain [40] and may reflect an ongoing role for sAPP in supporting neuron function in the context of β-amyloid toxicity, neuron stress, and inflammatory responses [34]–[38]. In the context of the greater overall stability of sAPP in the Tg2576 mice, substantial differences were also seen in the turnover of the specific sAPP species, with the murine sAPPα the most stable of the sAPP species. Given that the Swedish mutation in the transgene human APP drives β-cleavage and sAPPβ production [41], [42], the disproportionate levels of sAPPβ in the Tg2576 mice may impact the turnover of the relatively underrepresented sAPPα. Additionally, Aβ pathology may alter the turnover of sAPPα through an interaction in the brain parenchymal between the sequence homology at the C-terminus of sAPPα with the N-terminus of Aβ. Additionally, overexpression of sAPP in the Tg2576 mouse may overload the degradation pathway, although the differences in the metabolism between human and murine sAPP species, with murine sAPPα showing the longest half-life, suggests that species differences and/or differences in the handling of the Swedish-mutation-containing sAPP fragments also play a role in brain sAPP turnover in the β-amyloid depositing mouse model.

We find that endogenous Aβ, both Aβ40 and Aβ42, is rapidly degraded following cycloheximide treatment and the depletion of pre-existing APP. Following γ-secretase inhibition, Aβ microdialysis measurements have given a half-life for extracellular Aβ of ∼2 hours [13], and Savage et al. [14] have shown that shifting APP processing to the α-secretase pathway resulted in a decline in total brain Aβ with a half-life between 1 and 2.5 hours. Our findings, which used a different experimental approach that does not specifically alter an APP cleavage step, further support the conclusion that Aβ is rapidly cleared from the normal brain. Soluble Aβ in the β-amyloid plaque containing Tg2576 mice showed a similar and rapid decrease following cessation of protein synthesis, again with Aβ40 and Aβ42 turnover being similar. These findings indicate that the soluble Aβ pool in a brain containing β-amyloid plaque is similarly accessible to degradation as is soluble Aβ in a normal brain. The reduction plateau reached in the Tg2576 mice for soluble Aβ, which is likely to be in equilibrium with plaque-associated Aβ, may represent additional DEA-extractable Aβ being liberated from β-amyloid plaques over time in the brain. While brain APP is metabolized rapidly in both wild-type and an APP transgenic mice, our findings unexpectedly show that sAPP is more stable *in vivo* in the β-amyloid containing Tg2576 brain when compared to wild-type mice. Additionally, the rapid turnover of a soluble pool of Aβ is similar in the wild-type and the Tg2576 mice in spite of the significant accumulation of plaque-associated Aβ in the brains of the transgenic mice. The slower turnover of sAPP in the mice with plaque pathology suggests that this neurotrophic APP metabolite may have an important role in the β-amyloid containing brain.

## Materials and Methods

### Cycloheximide treatment

All experiments involving mice received prior approval from the Nathan Kline Institute Animal Care and Use Committee. Sixteen-month-old Tg2576 mice (overexpressing Swedish-mutant human APP) [15] and wild-type littermates on a Swiss Webster x DBA/C57BL6 F1 background were injected ip with 4.8 mg cycloheximide in 150 µl isotonic saline (containing 20 mM HEPES, pH 8), which has been shown to block protein synthesis in the brain of mice [21]. Mice were returned to their home cage, maintained at room temperature (22°C), sacrificed at the times indicated, and brain tissue was dissected and flash-frozen prior to storage at −80°C. The quantification of Western blot band density in Figure 1B, 1E, 1F, 1G and 3A is from two experiments with a total for both wild-type and Tg2576 of three mice at 0 and 23 hours cycloheximide treatment, two mice at 2, 4 and 6 hours treatment and one mouse at 1, 3, 5 and 7 hours treatment. In the tau experiments shown in Figure 3B, half of the mice were held in their home cage at a temperature of 34°C following cycloheximide injection.

### Cell cultures

Primary rat neuronal cultures were established from E18 embryos as previously described [43], and maintained in Neurobasal Media (minus L-glutamine and phenol red) containing B27 supplement, penicillin-streptomycin and Gluta MAX-1 (all from Invitrogen, Carlsbad, CA) for 10 days. Primary neurons were treated with 40 µg of cycloheximide per 1 ml media for the times indicated prior to lysis and recovery of proteins for Western blotting as previously described [44].

### Antibodies

Antibodies C1/6.1 and m3.2 were generated in our laboratory: C1/6.1 recognizes the carboxyl-terminal cytoplasmic domain of APP [44], while the epitope for m3.2 has been determined to be within residues 10–15 of murine Aβ by mapping against synthetic peptides m3.2 recognizes murine APP, sAPPα and Aβ (See Figure S1). 22C11 was purchased from Millipore (Temecula, CA) and recognizes an N-terminal APP epitope common to the human and murine proteins. Monoclonal antibody 6E10 (Covance, Princeton, NJ), which recognizes residues 1–16 of human Aβ, was used to detect human sAPPα. Rabbit polyclonal anti-sAPPβ antibody 242 recognizes only wild-type (murine or human) sAPPβ but not Swedish-mutant-derived sAPPβ [45], and mouse monoclonal antibody 6A1 recognizes sAPPβ containing the Swedish mutation but not the wild-type sequence [46]. These sAPP antibodies (242, 6A1) recognize cleaved sAPP fragments and are not cross-reactive with uncleaved APP. Total tau protein was detected by a phosphate-independent anti-tau monoclonal antibody (Tau T57120; BD Bioscience, San Jose, CA). PHF-1 recognizes tau protein phosphorylated at serine residues 396 and 404 [47]. Neprilysin was detected with the monoclonal antibody 56C6 (CD10) (Novacastra, Newcastle, UK), and IDE with the rabbit polyclonal antibody IDE1 ([11]; a gift of Dr. Dennis Selkoe).

### Brain Processing, Western blotting, Detection of APP Metabolites

Ten-percent (weight-to-volume) homogenates were prepared (in 250 mM sucrose, 20 mM Tris base, 1 mM EDTA, 1 mM EGTA and protease inhibitors) from hemibrains lacking the olfactory bulb and cerebellum [48], and these homogenates were subsequently used for Western blotting and extraction prior to Aβ sandwich ELISA. For Western blot analysis, membranes were incubated in the appropriate primary antibody (2 µg/ml) overnight and HRP-coupled secondary antibody for 1 hour.

### Aβ sandwich ELISAs

Murine Aβ levels were determined by sandwich ELISA using aliquots of the 10% sucrose homogenate. Soluble Aβ from an aliquot of the sucrose homogenates of wild-type and Tg2576 mice was extracted by diethylamine (DEA) [48]; separately, formic-acid extraction of another aliquot of the sucrose homogenate was used to detect β-amyloid plaque-associated Aβ in the Tg2576 mice. In combination with Aβ40 and Aβ42 C-terminal specific monoclonal antibodies (capture antibodies JRF/cAβ40/10 and JRF/cAβ42/26) [49], horseradish peroxidase-conjugated m3.2 was used to detect endogenous murine Aβ in Aβ40- and Aβ42-specific sandwich ELISAs [50]. Similar ELISAs using JRF/AβN25 [51] were used to detect human Aβ in the Tg2576 mouse brains. ELISA results are reported as the mean ± SEM in fmol Aβ per g wet brain, based on standard curves using synthetic murine and human Aβ1−40 and Aβ1−42 peptide standards (American Peptide).

### Statistics

Western blots were quantitated using ImageJ (http://rsb.info.nih.gov) and data analysis and graphs were plotted on GraphPad Prism v.5 (GraphPad Software, San Diego, CA, USA). Curve fitting and statistical two-way analysis of variance (ANOVA) were performed.

## Supporting Information

Figure S1 Characterization of the monoclonal antibody m3.2. Antibody m3.2, which was generated in our laboratory against a synthetic peptide corresponding to residues 1–15 of murine Aβ, binds specifically to murine APP, sAPPα and Aβ. A. Specificity of m3.2 antibody for murine Aβ compared to human Aβ shown by immunoprecipitation and Western blotting. Equal amounts (3 µg) of synthetic murine Aβ42 and human Aβ42 were immunoprecipitated overnight [44] with m3.2 and 6E10 antibodies as indicated. The immunoprecipitate was resolved by SDS-PAGE, transferred to membrane, and probed with m3.2 antibody. Murine Aβ was detected in the m3.2 immunoprecipitation of synthetic murine Aβ42; human Aβ was not detected. IgG heavy and light chain reactivity is with the secondary detection antibody. B. Human Aβ was immunoprecipitated by antibody 6E10 and not detected by antibody m3.2. As was done in S1A, synthetic human and murine Aβ were immunoprecipitated as indicated. Human Aβ was immunoprecipitated and detected by antibody 6E10, while antibody m3.2 showed no reactivity for the human Aβ. C. Specificity of antibody m3.2 for murine APP metabolites. Western blotting using antibody m3.2 of human control and AD brain and wild-type and aged Tg2576 mouse brain is shown. Consistent with peptide mapping showing that the m3.2 antibody epitope is within residues 10–15 of murine Aβ (data not shown), antibody m3.2 detected murine APP and co-migrating sAPPα in the mouse brain extract, but did not detect any proteins in the human brain extracts. Additionally, antibody m3.2 detected the abundant murine Aβ co-deposited in aged Tg2576 mouse brain. D. Antibody m3.2 reactivity with mouse brain extract is blocked by co-incubation with synthetic murine Aβ. Wild-type and aged Tg2576 mouse brain extracts were resolved by SDS-PAGE as indicated. Synthetic murine Aβ42 or synthetic human Aβ was added to the antibody m3.2 binding solution as indicated (1 µg/ml murine or human Aβ, 2 µg/ml m3.2 antibody) 1 hour prior to membrane incubation. APP metabolites were not detected with the addition of competing synthetic murine Aβ42. The addition of synthetic human Aβ42 did not interfer with m3.2 reactivity with murine APP metabolites in the mouse brain. E. Antibody m3.2 detects both APP and sAPPα in mouse brain. Mouse brain homogenates were subjected to centrifugation at 100,000×g for 1 hour as previously described [48]. Equivalent amounts of the membrane pellet containing APP and the supernatant containing soluble sAPP were resolved by SDS-PAGE and probed with either antibody m3.2 or 22C11 as indicated. The subtle mobility shift in the smaller sAPP species is shown for sAPPα (m3.2) and total sAPP (22C11) compared to the membrane-associated APP in the pellet fractions. These Western blots also demonstrate the steady-state abundance of sAPPα (m3.2) and total sAPP (22C11) relative to APP, consistent with the slower turnover of sAPP in the mouse brain.
(9.70 MB TIF)
