# Supplementary figures and images for: In Vivo Turnover of Tau and APP Metabolites in the Brains of Wild-Type and Tg2576 Mice: Greater Stability of sAPP in the β-Amyloid Depositing Mice
Source: PLoS One. 2009 Sep 22;4(9):e7134. doi: 10.1371/journal.pone.0007134 (PMC2741602; doi:10.1371/journal.pone.0007134)

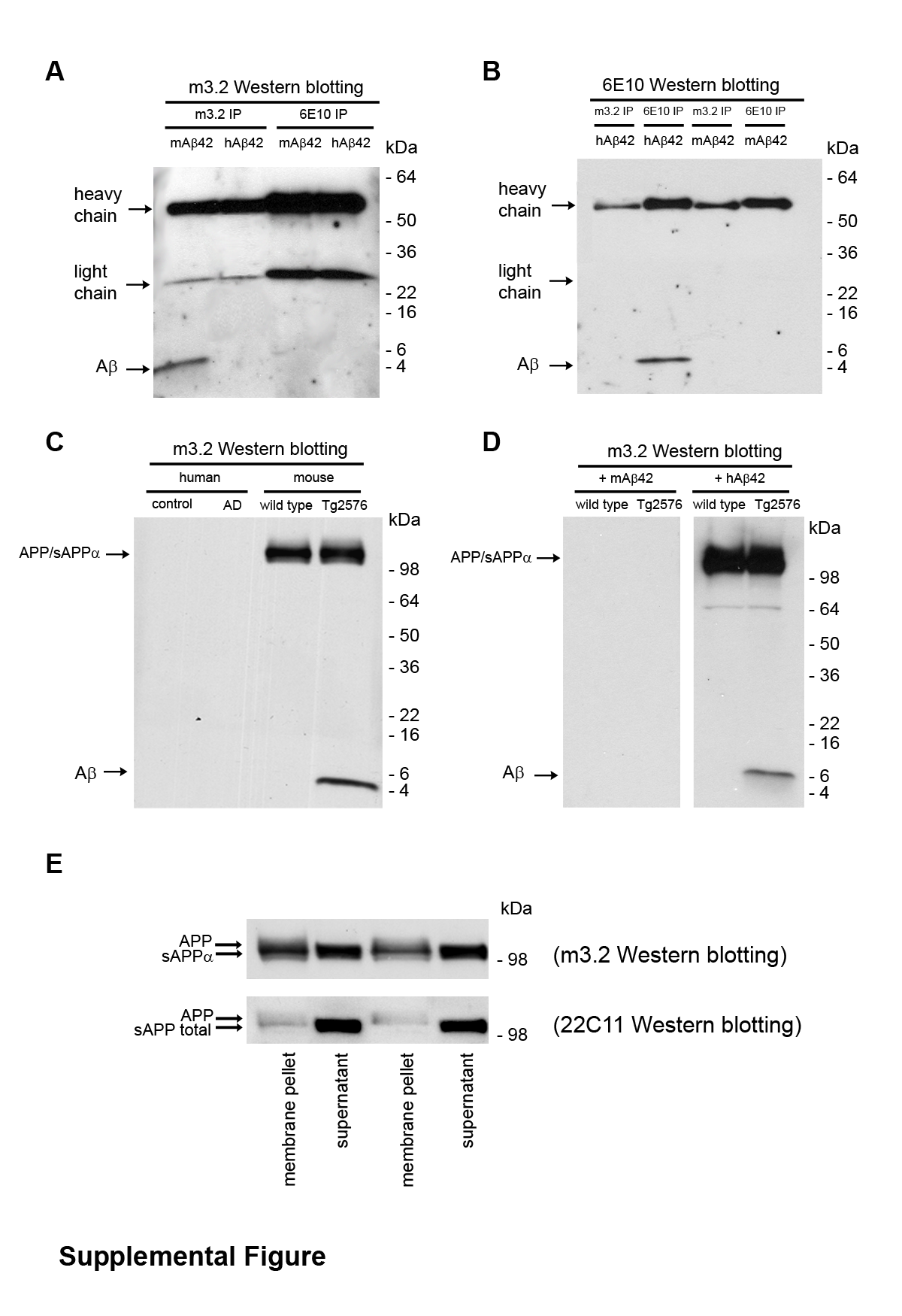

Supplement: Figure S1 — Characterization of the monoclonal antibody m3.2. Antibody m3.2, which was generated in our laboratory against a synthetic peptide corresponding to residues 1–15 of murine Aβ, binds specifically to murine APP, sAPPα and Aβ. A. Specificity of m3.2 antibody for murine Aβ compared to human Aβ shown by immunoprecipitation and Western blotting. Equal amounts (3 µg) of synthetic murine Aβ42 and human Aβ42 were immunoprecipitated overnight [44] with m3.2 and 6E10 antibodies as indicated. The immunoprecipitate was resolved by SDS-PAGE, transferred to membrane, and probed with m3.2 antibody. Murine Aβ was detected in the m3.2 immunoprecipitation of synthetic murine Aβ42; human Aβ was not detected. IgG heavy and light chain reactivity is with the secondary detection antibody. B. Human Aβ was immunoprecipitated by antibody 6E10 and not detected by antibody m3.2. As was done in S1A, synthetic human and murine Aβ were immunoprecipitated as indicated. Human Aβ was immunoprecipitated and detected by antibody 6E10, while antibody m3.2 showed no reactivity for the human Aβ. C. Specificity of antibody m3.2 for murine APP metabolites. Western blotting using antibody m3.2 of human control and AD brain and wild-type and aged Tg2576 mouse brain is shown. Consistent with peptide mapping showing that the m3.2 antibody epitope is within residues 10–15 of murine Aβ (data not shown), antibody m3.2 detected murine APP and co-migrating sAPPα in the mouse brain extract, but did not detect any proteins in the human brain extracts. Additionally, antibody m3.2 detected the abundant murine Aβ co-deposited in aged Tg2576 mouse brain. D. Antibody m3.2 reactivity with mouse brain extract is blocked by co-incubation with synthetic murine Aβ. Wild-type and aged Tg2576 mouse brain extracts were resolved by SDS-PAGE as indicated. Synthetic murine Aβ42 or synthetic human Aβ was added to the antibody m3.2 binding solution as indicated (1 µg/ml murine or human Aβ, 2 µg/ml m3.2 antibody) 1 hour prior to membra [file pone.0007134.s001.tif]
